# Laparoscopic Splenectomy to Salvage Renal Transplants from Severe Acute Antibody-Mediated Rejection

**DOI:** 10.1155/2012/253173

**Published:** 2012-12-18

**Authors:** Michael Latzko, Sakshi Jasra, Sana Akbar, Harry Sun, Sadanand Palekar

**Affiliations:** Department of Renal Transplantation and Department of Surgery, Newark Beth Israel Medical Center, Newark, NJ 07112, USA

## Abstract

* Purpose*. Acute antibody-mediated rejection, a complication of cross match positive and sensitized renal transplants, occurs despite the use of standard desensitization protocols. Rescue therapy consists of plasmapheresis and intravenous immunoglobulin (IVIg). In patients with preformed donor specific antibodies, rejection can be aggressive. We report here a case in which laparoscopic splenectomy was added to the standard rescue regimen. *Case Report and Results*. A 40-year-old Hispanic female with end stage renal disease had been receiving hemodialysis. The patient had numerous class 1 unacceptable antigens. She was scheduled to undergo an incompatible 1-1-1 mismatch living related donor kidney transplant. Preoperatively, the patient received plasmapheresis, IVIG, and thymoglobulin. There was good graft function until postoperative day 5. At that point, worsening renal function was noted. Renal biopsy was consistent with AMR. The patient became anuric and dialysis was initiated. To salvage the transplant, the patient underwent laparoscopic splenectomy. Postoperatively, renal function improved. Two years after transplant, the patient continues to have excellent graft function. *Conclusion*. In a small but significant number of renal transplants, antibody production occurs at a rate that traditional treatments are unable to reduce effectively. Based on our experience, the addition of splenectomy to standard rescue therapy can salvage renal transplants.

## 1. Introduction

The recent development of desensitization protocols that utilize a combination of plasmapheresis (PP) and intravenous immunoglobulins (IVIg) has expanded the indications for living donor kidney transplantation to include HLA and ABO-incompatible recipients. Although graft survival has been shown to improve with the introduction of these therapies, the incidence of antibody-mediated rejection (AMR) remains high, and has proved to be a difficult barrier to overcome [1–5]. AMR is characterized by (a) increasing donor specific antigen (DSA) titers, (b) allograft dysfunction, as demonstrated by declining creatinine clearance, and (c) deposition of C4d in peritubular capillaries [6]. Clinically, all three criteria may not always be present and do not need to be met in order to definitively diagnose AMR.

In the last decade, several new therapies have emerged that attempt to treat AMR. However, there remain a small but significant percentage of cases that are refractory to classical treatments [6]. Rescue splenectomy has been proposed as a last salvage option for such cases. The spleen acts as a repository for memory B cells and plasma cells, thus playing an important role in the alloantibody response. To our knowledge, a very limited number of cases have been reported where splenectomy has been effectively utilized in the postoperative period as a rescue therapy. We report here results obtained over a period of two years in a patient who underwent splenectomy after developing AMR after transplant and remaining refractory to the standard desensitization protocols available at the time. 

## 2. Case Report

A 40-year-old Hispanic female with a past medical history significant for end stage renal disease (ESRD) on hemodialysis (HD) was referred to the Newark Beth Israel Renal Transplant Program for the evaluation for a living donor kidney transplant. The patient had numerous unacceptable class 1 antigens with a historic panel reactive antibody (PRA) titer of 96%. At the time of transplantation, the final complement dependent cytotoxicity (CDC) cross-match results were positive by NEAT and negative by dithiothreitol (DTT), with positive B and T cell flow cross-matches with mean channel shift (MCS) values of 423 and 524.5, respectively. There were two class 1 DSAs present, with an elevated mean MFI of 1247. After induction therapy with PP and IVIg as per protocol, the patient received a live donor renal transplant from her nephew.

Immunosuppressive therapy at the time of transplantation consisted of induction with Thymoglobulin given over five days for a total dosage of 6 mg/kg. Corticosteroids were also initiated intraoperatively (Solu-medrol 500 mg every 12 hours for 3 days with a prednisone taper). Mycophenolate mofetil 1.0 g twice a day was also started on the first postoperative day. In addition, tacrolimus was started at 0.1 to 0.2 mg/kg twice a day with dosages adjusted to keep serum trough levels at 8–15 ng/mL. Postoperatively, there was immediate graft function with normalization of serum creatinine and good urine output. However, on postoperative day 5, there was a marked decrease in urine output, worsening renal function and rising DSA titers with a mean MFI of 11636. A renal transplant biopsy done at this time showed evidence of fibrin thrombi, cellular changes consistent with AMR, and C4d positive staining. During this interval, the patient was continued on PP, IVIg, Cytogam, and maintenance transplant immunosuppressives of mycophenolate mofetil, steroids, and tacrolimus. Despite the aforementioned measures, the patient subsequently became anuric and needed dialysis. 

**Figure 1:**
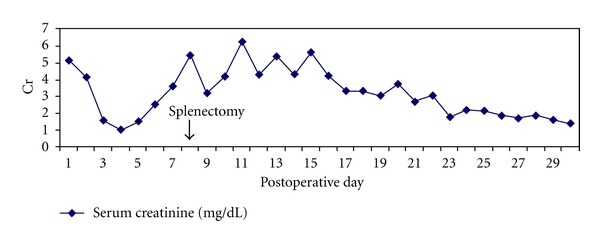


 Due to the marked decline in the renal function, the decision was made to perform a rescue laparoscopic splenectomy. The patient tolerated the procedure well with no intraoperative complications. The postoperative course was briefly complicated by an upper gastrointestinal bleed secondary to a gastric ulcer, successfully managed with endoscopic cauterization. Within one week of undergoing splenectomy, urine output was found to improve significantly, with decreasing serum creatinine levels (Figure 1). Repeat antibody screening revealed serially decreasing DSA levels with a mean MFI of 4132 at one month postoperatively. At present, the patient is at two years after transplant and continues to have excellent graft function.

## 3. Discussion

 In recent years, newer methods of immunosuppression have emerged that help broaden the scope of renal transplants. However, the high burden of anti-HLA DSAs that may be present in a small, but significant, number of patients continues to be a challenge [7]. Such antibodies may form in patients who have previously been sensitized, either by blood transfusions, other organ transplants, or even via pregnancy [8]. Traditionally, plasmapharesis along with IVIg has been used to control DSA titers in the immediate postoperative period [6]. Although there is some data available on the utility of splenectomy as a preemptive measure before titers increase, this may be associated with an increase in mortality. Thus, most centers have resorted to complying with the original regimen of intensive peritransplantation PP in combination with IVIg [9–11]. We present here a unique case of a highly sensitized adult patient who developed severe AMR after renal transplant that was refractory to PP, IVIg, steroids, and Thymoglobulin treatment. 

To our knowledge, there are only two case reports conducted in adults, and one pediatric case report, that show a benefit of splenectomy as a rescue measure post-transplant [5, 7, 12]. The spleen acts to generate and store plasma cells and memory B cells [13]. Pathological examinations of spleens removed from patients who suffered acute severe AMR have demonstrated the presence of abundant numbers of mature B cells and CD138+ plasma cells [14]. Studies show that while under severe antigenic stress, the spleen can induce the rapid differentiation of B cells into antigen producing plasma cells [14]. In addition, the role of the spleen in acting as a store for IgM memory cells has been well established [7]. Memory lymphocytes can be activated quickly and thus make up the basis for a rapid immune response to alloantigen [7]. In such a scenario, the burden of antibodies can quickly increase to a level that traditional regimens of PP/IVIg are unable to fully tackle [5].

 Recently, newer immunosuppressants in the form of anti-CD20 antibody (Rituximab) and anticomplement antibody (Eculizumab) have been proposed as additional therapies to treat AMR [15]. Although these can be used as an adjuvant to control antibody titers, splenectomy still remains the quickest and most efficient means of significantly decreasing titers to a level that can actually salvage the transplant. Thus, by removing the spleen, the bulk of antibody generation is removed, allowing PP and IVIg to be more effective. There is data suggesting that splenectomy alone can lead to rapid diuresis and immediate restoration of renal function, although the exact mechanism remains unknown [5]. This improvement may even occur prior to the reinitiation of PP, further highlighting the importance of splenectomy in the debulking of plasma cells [5]. Similarly, in our patient, splenectomy was able to fully reverse the refractory acute AMR and restore urine output within one week. Two years after transplant, the patient continues to have excellent graft function. 

It is important to mention that given that the CD20 cell marker is not actively produced by plasma cells, anti-CD20 antibody is unlikely to be successful in preventing further generation of antibodies in the acute setting [5]. It may, however, have some benefit preoperatively in preventing the formation of plasma cells that can mediate AMR by blocking pre-B cells and B cells [5]. Similarly, the utility of anticomplement antibodies has also not been proven as a replacement therapy to splenectomy, but simply as an additional treatment. Although there is some concern regarding the increased risk of postsplenectomy sepsis, careful management of the patient with appropriate vaccines preoperatively, antibiotics, along with a more focused regimen of immunosuppression can help decrease this risk [5]. 

In conclusion, we propose the addition of splenectomy in the regimen to treat persistent allograft dysfunction in patients that have already undergone treatment with PP/IVIg. Further studies in terms of randomized controlled trials need to be conducted in order to fully delineate and confirm our finding, but emergent splenectomy appears to be an excellent option to effectively deplete circulating memory B cells and plasma cells, thereby decreasing antibody production to a level that can be controlled with the traditional regiment of PP/IVIg.

## References

[1] Gloor JM, DeGoey SR, Peneda AA (2003). Overcoming a positive crossmatch in living-donor kidney trasnplantation. *American Journal of Transplantation*.

[2] Montgomery RA, Zachary AA (2004). Transplanting patients with a positive donor-specific crossmatch: a single center’s perspective. *Pediatric Transplantation*.

[3] Montgomery RA, Zachary AA, Racusen LC (2000). Plasmapheresis and intravenous immune globulin provides effective rescue therapy for refractory humoral rejection and allows kidneys to be successfully transplanted into cross-match-positive recipients. *Transplantation*.

[4] Aikawa A, Ohara T, Arai K (2004). Clinical outcome and accommodation in ABO incompatible kidney transplantation. *Clinical Transplants*.

[5] Locke JE, Zachary AA, Haas M (2007). The utility of splenectomy as rescue treatment for severe acute antibody mediated rejection. *American Journal of Transplantation*.

[6] Fehr T, Gespart A (2012). Antibody-mediated kidney allograft rejection: therapeutic options and their experimental rationale. *Transplant International*.

[7] Roberti I, Geffner S, Vyas S (2012). Successful rescue of refractory acute antibody-mediated renal allograft rejection with splenectomy—a case report. *Pediatric Transplantation*.

[8] Monteiro F, Buelow R, Mineiro C, Rodrigues H, Kalil J (1997). Identification of patients at high risk of graft loss by pre- and posttransplant monitoring of anti-HLA class I IgG antibodies by enzyme-linked immunosorbent assay. *Transplantation*.

[9] Akalin E, Dinavahi R, Friedlander R (2008). Addition of plasmapheresis decreases the incidence of acute antibody-mediated rejection in sensitized patients with strong donor-specific antibodies. *Clinical Journal of the American Society of Nephrology*.

[10] Alexander JW, First MR, Majeski JA (1984). The late adverse effect of splenectomy on patient survival following cadaveric renal transplantation. *Transplantation*.

[11] Sawada T, Fuchinoue S, Kawase T, Kubota K, Teraoka S (2004). Preconditioning regimen consisting of anti-CD20 monoclonal antibody infusions, splenectomy and DFPP-enabled non-responders to undergo ABO-incompatible kidney transplantation. *Clinical Transplantation*.

[12] Kaplan B, Gangemi A, Thielke J, Oberholzer J, Sankary H, Benedetti E (2007). Successful rescue of refractory, severe antibody mediated rejection with splenectomy. *Transplantation*.

[13] Nolte M, Hoen E, van Stijin A (2000). Isolation of the intact white pulp. Quantitative and qualitative analysis of the cellular composition of the splenic compartments. *European Journal of Immunology*.

[14] Kaplan B, Jie T, Diana R (2010). Histopathology and immunophenotype of the spleen during acute antibody-mediated rejection. *American Journal of Transplantation*.

[15] Stewart ZA, Collins TE, Schlueter AJ Case report: eculizumab rescue of severe accelerated antibody-mediated rejection after ABO-incompatible kidneytransplant.

